# Genomic features, antimicrobial susceptibility, and epidemiological insights into *Burkholderia cenocepacia* clonal complex 31 isolates from bloodstream infections in India

**DOI:** 10.3389/fcimb.2023.1151594

**Published:** 2023-04-19

**Authors:** Tanu Saroha, Prashant P. Patil, Rekha Rana, Rajesh Kumar, Sanjeet Kumar, Lipika Singhal, Vikas Gautam, Prabhu B. Patil

**Affiliations:** ^1^ Bacterial Genomics and Evolution Laboratory, Council of Scientific and Industrial Research (CSIR)-Institute of Microbial Technology, Chandigarh, India; ^2^ Academy of Scientific and Innovative Research (AcSIR), Ghaziabad, India; ^3^ Department of Medical Microbiology, Post Graduate Institute of Medical Education and Research, Chandigarh, India; ^4^ Department of Microbiology, Government Medical College and Hospital, Chandigarh, India

**Keywords:** *Burkholderia cenocepacia* clonal complex 31, sequence types, evolution, antibiotic resistance, mobile genetic elements, virulence genes, pangenomics

## Abstract

**Introduction:**

*Burkholderia cepacia* complex (Bcc) clonal complex (CC) 31, the predominant lineage causing devastating outbreaks globally, has been a growing concern of infections in non-cystic fibrosis (NCF) patients in India. *B. cenocepacia* is very challenging to treat owing to its virulence determinants and antibiotic resistance. Improving the management of these infections requires a better knowledge of their resistance patterns and mechanisms.

**Methods:**

Whole-genome sequences of 35 CC31 isolates obtained from patient samples, were analyzed against available 210 CC31 genomes in the NCBI database to glean details of resistance, virulence, mobile elements, and phylogenetic markers to study genomic diversity and evolution of CC31 lineage in India.

**Results:**

Genomic analysis revealed that 35 isolates belonging to CC31 were categorized into 11 sequence types (ST), of which five STs were reported exclusively from India. Phylogenetic analysis classified 245 CC31 isolates into eight distinct clades (I-VIII) and unveiled that NCF isolates are evolving independently from the global cystic fibrosis (CF) isolates forming a distinct clade. The detection rate of seven classes of antibiotic-related genes in 35 isolates was 35 (100%) for tetracyclines, aminoglycosides, and fluoroquinolones; 26 (74.2%) for sulphonamides and phenicols; 7 (20%) for beta-lactamases; and 1 (2.8%) for trimethoprim resistance genes. Additionally, 3 (8.5%) NCF isolates were resistant to disinfecting agents and antiseptics. Antimicrobial susceptibility testing revealed that majority of NCF isolates were resistant to chloramphenicol (77%) and levofloxacin (34%). NCF isolates have a comparable number of virulence genes to CF isolates. A well-studied pathogenicity island of *B*. *cenocepacia*, GI11 is present in ST628 and ST709 isolates from the Indian Bcc population. In contrast, genomic island GI15 (highly similar to the island found in *B*. *pseudomallei* strain EY1) is exclusively reported in ST839 and ST824 isolates from two different locations in India. Horizontal acquisition of lytic phage ST79 of pathogenic *B*. *pseudomallei* is demonstrated in ST628 isolates Bcc1463, Bcc29163, and BccR4654 amongst CC31 lineage.

**Discussion:**

The study reveals a high diversity of CC31 lineages among *B. cenocepacia* isolates from India. The extensive information from this study will facilitate the development of rapid diagnostic and novel therapeutic approaches to manage *B*. *cenocepacia* infections.

## Introduction

*Burkholderia cepacia* complex (Bcc) contains 22 validly published species of non-fermenting gram-negative bacilli (NFGNB) (Jin et al., 2020). Members of Bcc are the fourth most common pathogenic NFGNB worldwide after *Acinetobacter*, *Pseudomonas*, and *Stenotrophomonas* in humans (Gautam et al., 2011).

Among Bcc members, *Burkholderia cenocepacia* and *Burkholderia multivorans* together account for the majority (85-97%) of clinical infections (Drevinek and Mahenthiralingam, 2010). *B*. *cenocepacia* has emerged as an important opportunistic pathogen in immunocompromised, cystic fibrosis (CF), and chronic granulomatous disease patients (Mahenthiralingam et al., 2005; Mahenthiralingam et al., 2008; Gautam et al., 2011). In patients with CF, they cause fatal cepacia syndrome characterized by a rapid decline of lung function with necrotizing pneumonia leading to bacteraemia, septicaemia, and increased mortality (Lee et al., 2017; Leitão et al., 2017). *B. cenocepacia* is prevalent in anthropogenic and natural environments as they have high capability for rapid mutation and metabolic diversity (Coenye and Vandamme, 2003; Mahenthiralingam et al., 2005). The ecological, genetic, and metabolic diversity of these bacteria is likely due to the size of their unusually large genome (7-9 Mbp) with multiple chromosomes, as well as their ability to utilize a wide range of compounds as sole carbon sources such as penicillin G. The frequent acquisition of horizontally transferred mobile genetic elements contributes to multi-drug resistance phenotype (Beckman and Lessie, 1979; Agnoli et al., 2014; Guo et al., 2017).

*B. cenocepacia* is reported to cause rampant outbreaks throughout the world (Sun et al., 1995; Zlosnik et al., 2015; Nunvar et al., 2017). During the 1990s, epidemic *B. cenocepacia* electrophoretic type 12 (ET12) lineage caused fatal and devastating infections in CF patients intercontinentally. ET12 [sequence type (ST)28] lineage is one of the most virulent and resistant Bcc studied until date (Holden et al., 2009; Ratjen and VanDevanter, 2022). Other than ET12 lineage, ST32 strains caused devastating health infections in Czech republic CF patients with wide distribution across several countries (Drevinek et al., 2010; Nunvar et al., 2017). Among *B. cenocepacia*, two lineages were defined on basis of *recA* gene similarity: IIIA [belongs to clonal complex (CC)31] and IIIB (Mahenthiralingam et al., 2000). CC31 is the largest cluster in Bcc multilocus sequence type (MLST) database corresponding to its frequent isolation from CF patients (Gautam et al., 2016). Previous research studies have established the destructive nature of IIIA lineage clones in epidemic outbreaks (Drevinek and Mahenthiralingam, 2010; Nunvar et al., 2017). They are identified by the presence of the *cblA* gene encoding giant cable pilus and *B. cepacia* epidemic strain marker (BCESM) (Van Acker et al., 2014). Genome sequencing revealed the acquisition of >10% *B. cenocepacia* genome through horizontal gene transfer contributing to their genome plasticity (Holden et al., 2009). There are fifteen genomic islands (GI1 to GI15) including one from our previous study identified in *B. cenocepacia* and amongst them, GI11 and GI15 encode genes that are involved in pathogenesis (Holden et al., 2009; Guo et al., 2017; Patil et al., 2017). Therapeutic options are limited for *B. cenocepacia* because they have intrinsic resistance to numerous classes of antibiotics, including aminoglycosides and polymyxins (Tseng et al., 2014; Abbott and Peleg, 2015). The genomes of *B. cenocepacia* are reported to have all five major classes of efflux pumps with the majority of them belonging to the RND (resistance-nodulation-cell division) type (Podnecky et al., 2015). Although *B. cenocepacia* CC31 isolates are reported from various countries such as Canada, the UK, the USA, and Europe, the present study has provided first detailed insights into genomic features of the CC31 isolates from non-cystic fibrosis (NCF) patients in India.

We analyzed the genomic features of 35 isolates belonging to 11 different STs in CC31 collected from India between 2005 and 2013. To delineate the genomic features and probable transmission of CC31 isolates, we selected additional 210 CC31 isolates from the NCBI GenBank database. Further, we carried out a phylogenetic analysis to construct the evolutionary dynamics of the CC31 lineage. Additionally, we performed the mobile genetic elements (MGE), resistome, virulome, and pan-genome analysis of the CC31 isolates. The genomic analysis will underline the genetic diversity, evolutionary relationships, and variations in MGE, resistome, and virulome repertoire in the CC31 lineage, which have important implications in the treatment, epidemiology, and management of this important group of pathogens.

## Materials and methods

### Collection, whole genome sequencing, annotation, and *in silico* MLST profiling of *B. cenocepacia* isolates

55 Bcc isolates were collected from routine patient specimens over 9 years (2005-2013) and identified using biochemical methods and matrix-assisted laser desorption-time of flight mass spectrometry MALDI TOF-MS (Bruker Corporation Ltd). 49 *B. cenocepacia* isolates were grown on Mueller-Hinton agar plates at 37°C for 16 hours and genomic DNA was isolated using Quick DNA bacterial/fungal miniprep kit (Zymo Research, USA). Genomic DNA contamination with RNA and protein was assessed by using NanoDrop (Thermo Scientific, USA) and quantified using Qubit 2.0 Fluorometer (Invitrogen; Thermo Fisher Scientific, USA). Contamination-free DNA was sequenced using the Illumina HiSeq X10 platform with a read length of 2×150 bp (AgriGenome Labs Pvt. Ltd, Kerala, India). The quality of reads was checked using FASTqc v0.11.9 (FastQC, 2020), reads trimmed using Trimgalore 0.6.6 (Krueger, 2017) were assembled *de novo* using SPAdes v3.13.0 (Bankevich et al., 2012). Assembled genome quality was checked using checkm v1.1.3 (Parks et al., 2015) considering cut-off values for completeness >99% and contamination < 2%. Further, assembly statistics were generated using QUAST v5.0.2. 6 (Gurevich et al., 2013) and BBMap version 38.87 (Bushnell, 2014). 6 out of 49 isolates were not included for further analysis due to poor quality of reads. The genomes submitted to NCBI with accession numbers are mentioned in **Table S2**. Annotation of genomes was performed using NCBI Prokaryotic Genome Annotation Pipeline 6.0. The ST information of isolates was retrieved from publicly available MLST v2.19.0 pipeline (https://github.com/tseemann/mlst), which uses the PubMLST database (www.pubMLST.org/bcc). The global relationship of STs in the MLST database was assessed using goeBURST v1.2.1 (Francisco et al., 2009). 35 out of 43 isolates belonging to different STs in CC31 were considered for further analysis. Additional genomes of CC31 isolates with geographic location, isolation source and year information were downloaded from the NCBI genome database and quality checked for completeness and contamination thresholds of 90% and 10% respectively using checkm v1.1.3 (Parks et al., 2015) (**Table S3**). A total of 245 (35 isolates from our collection and 210 as previously mentioned) genomes of CC31 were used in analysis.

### Antimicrobial susceptibility testing

The isolates from our collection were subjected to antimicrobial susceptibility testing by the Kirby-Bauer disk diffusion test according to recent Clinical Laboratory Standards Institute guidelines (CLSI). This test was done for cotrimoxazole (1.25 μg/23.75 μg), ceftazidime (30 μg), tetracycline (30 μg), levofloxacin (5 μg), meropenem (10 μg), minocycline (30 μg), Piperacillin-tazobactam (100μg/10 μg), and chloramphenicol (30 μg). Minimum inhibitory concentrations (MIC) were also determined by agar dilution as per CLSI guidelines against minocycline (susceptible, S ≤ 4 and resistant R≥16 μg/ml), levofloxacin (S ≤ 2 & R≥8 μg/ml), chloramphenicol (S ≤ 8 & R≥32 μg/ml), and ceftazidime (S ≤ 8 & R≥32 μg/ml).

### Taxonogenomic and phylogenomic assessment

Average nucleotide identity (ANI) was calculated by the standalone OrthoANI OAU v1.2 tool (Lee et al., 2016). ANI values of Bcc genomes with the genomes of the type strain of the *Burkholderia* genus were calculated to confirm the species status. The heat map of the ANI values between sequenced isolates and type strains of *Burkholderia* genus was constructed using TBtools v1.098726 (Chen et al., 2020). To further confirm species status, genomes were annotated using Prokka 1.14.6 (Seemann, 2014) and catenated alignment of 1418 core genes generated by Roary 3.13.0 (Page et al., 2015) with identity and maximum cluster number of 70% and 60000, respectively, was used for maximum likelihood phylogeny construction using PhyML 3.3.20200621 (Guindon et al., 2010).

### CC31 core SNP phylogeny

Snippy v4.6.0 (Seemann, 2019) was used to perform reference-based mapping in the CC31 core genome, with *Burkholderia cenocepacia* J2315 used as a reference strain. The putative recombination regions within the core genome were identified and masked using Gubbins 2.4.1 (Croucher et al., 2015) and a python script maskrc-svg v0.5 (https://github.com/kwongj/maskrc-svg), respectively. Further, the masked alignment was used as input to construct recombination-free maximum likelihood phylogeny with PhyML 3.3.20200621 (Guindon et al., 2010) using the GTR+GAMMA model, and 500 bootstrap replicates. The tree was visualized using iTOL v6 (Letunic and Bork, 2019) and R package ggtreeExtra 1.8.1 (Xu et al., 2021). The phylogenetic lineages in the tree were detected using R package r-fastbaps v1.0.4 (fast hierarchical Bayesian Analysis of Population Structure model) (Tonkin et al., 2019).

### Pan-genome analysis

The pan-genomic analysis of 35 CC31 Indian isolates with *B. cenocepacia* J2315 as reference strain was performed using anvi’o platform v7.1 (Eren et al., 2021) following the instructions in the microbial pan- genomics tutorial. Briefly, genes were predicted using Prodigal, proteins were annotated with Clusters of Orthologous Groups (COGs; 2014 release), and protein families were identified using hidden markov model profiles. Additionally, the muscle tool was used for sequence alignment, the Markov cluster (MC) technique for clustering, and NCBI blastp to determine the similarity of amino acid sequences. The remaining parameters for calling gene clusters, such as MC inflation, minbit heuristics were set as default. Validation of pan-genome was carried out using roary 3.13.0 (Page et al., 2015) with default parameters. The pan-genome characteristic curve was drawn using roary 3.13.0 (Page et al., 2015).

### Resistome and virulome analysis of CC31 isolates

The AMR genes were analysed using Resistance Gene Identifier 5.2.0 implemented in Comprehensive Antibiotic Resistance Database 3.1.4 (Alcock et al., 2020). The heatmap of resistance genes was constructed using R v4.2.0 using the gheatmap function in ggtree 3.6.2 package (Yu et al., 2017).The nucleotide sequences of well-characterised 352 virulence genes identified in *B. cenocepacia* (Holden et al., 2009) were used as query in TBLASTN 2.12.0+ (Camacho et al., 2009) with the percent identity of 70% and query coverage of 75%. The heatmap depicting the presence and absence of virulence genes among the CC31 isolates was created using TBtools v1.098726 (Chen et al., 2020).

### Detection of genomic islands and phages

Fifteen genomic islands (GI1-GI15) reported in Bcc until date were analysed in 245 Bcc isolates. The nucleotide sequence of genomic islands (GI1 to GI14) in the genome of Bcc J2315 and BccIPCUA were extracted based on coordinates (Holden et al., 2009; Patil et al., 2017), and BLASTN (Camacho et al., 2009) analysis was carried out with a percent identity of 85% and a query coverage of 75%. The heatmap of the presence and absence of genomic islands was constructed using R v4.2.0 using the gheatmap function in the ggtree 3.6.2 package (Yu et al., 2017). Pathogenicity island GI11 of *B. cenocepacia* J2315 was used as a reference for comparison amongst CC31 isolates using clinker v0.0.23 (Gilchrist and Chooi, 2021).

The coordinates and annotation of phages in the CC31 isolates were fetched using the PHASTER server (Arndt et al., 2016). The GenBank files of 39 phages previously reported in the *Burkholderia* genus (Roszniowski et al., 2018) were extracted and compared to phages in CC31 isolates using clinker v0.0.23 with default settings (Gilchrist and Chooi, 2021). It performs protein sequence alignment and generates results based on sequence similarity of default 30% identity.

### SNPs detection

The differences in SNPs between the isolates of each ST were determined using snp-dists 0.8.2 tool (https://github.com/tseemann/snp-dists).

## Results

### Genome characteristics and identification of Indian Bcc isolates

Whole-genome sequencing of 43 Bcc isolates was performed in this study. The genome size ranged from 6.6Mbp to 8.6Mbp with GC content of around 66-67%. The number of coding sequences (CDS) in annotated genomes ranged from 6004 to 8185. The estimated genome completeness for this dataset is >99%, and the estimated contamination is <2% suggesting a high-quality draft genome. The assembly statistics are summarized in **Table S1**. The genomic features, sequence type, and accession numbers of 43 Bcc isolates are described in **Table S2**. For taxonomic identification of isolates, ANI was calculated where 95-96% cut-off was used for species delineation (Richter and Rosselló-Móra, 2009). Based on the cut-off values for ANI, the isolates sequenced belong to *B. cenocepacia* (**Figure S1A**). The species status was further confirmed by phylogenetic analysis, and the isolates form a separate clade clustering together with the type strain of *B. cenocepacia* NCTC 13227 (**Figure S1B**).

### Geographic distribution

We analyzed 245 *B. cenocepacia* CC31 isolates collected between 1985 and 2020 (210 from the NCBI database and 35 from our collection), spread across seven different countries in four continents (Asia, North and South America, Europe). The geographic location, isolation source, date of collection, and sequence type information are summarized in **Table S3**. Majority of isolates (n=197, 80% of the total) were isolated from CF patients. Out of which a large proportion of isolates were isolated from Canada (88%, 174/197) followed by the USA (8%, 16/197). Of the 245 CC31 isolates, 44 (18% of the total) isolates were isolated from NCF patients, mostly from blood. Isolates in our collection constitute 68% (30/44) isolates from the NCF population (**Table S3**). **Table S4** summarizes the number of isolates from each isolation source.

### Phylogenomics

**Figure 1:**
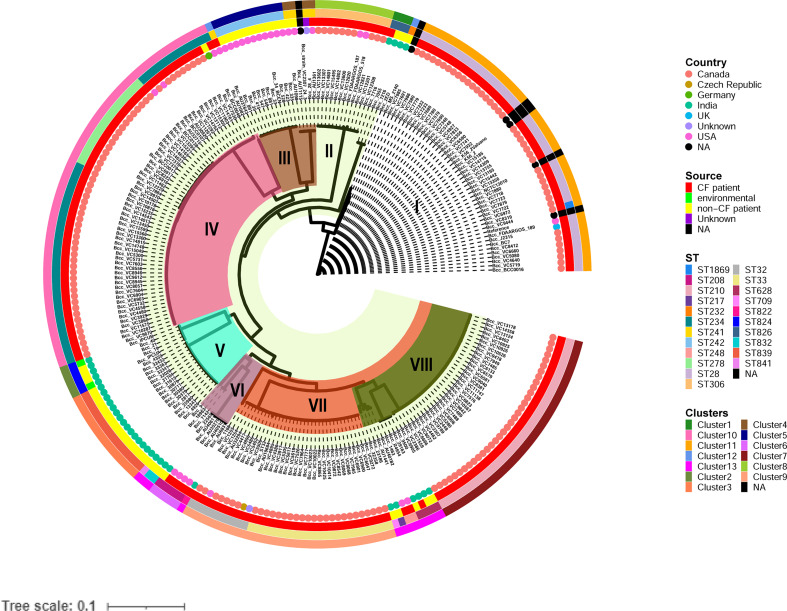
Phylogenetic structure of 245 CC31 isolates. Clades I-VIII are marked by background colour fill. Data on the country of isolation, source of isolation, MLST classification, and fastbaps clusters are depicted on the tree (from inner to outer circle).

To analyze the genetic and evolutionary divergence of the CC31 isolates, we constructed a maximum likelihood core single nucleotide polymorphism (SNP) phylogeny obtained from PhyML consisting of eight clades. Clade I, IV, and VII consisted predominantly of CF isolates, except one isolate (BccBCC0048) in clade IV and three isolates (Bcc7216, Bcc7055, and Bcc2ED) in clade I. In contrast, Clade III and V consisted of only NCF isolates from India (n=30) and USA (n=12). In addition, isolates collected from India and USA were distributed across four clades of the tree, indicating that isolates had greater variability in the core genomic regions (**Figure 1**). Correlation of source and clusters depicted in **Figure S2**.

The 245 CC31 isolates were arranged into thirteen clusters using the first level of clustering in fast hierarchical Bayesian analysis of population structure (fastbaps). The fastbaps clusters were in accordance with clades obtained from maximum-likelihood phylogeny and depicted in the fourth data strip of **Figure 1**. Fastbaps clusters 1, 2, and 3 belonged to the clade I, V consisting of only Indian NCF isolates, whereas clusters 5 belonged to clade III consisting of NCF isolates from the USA. Clusters 7-10 consisted entirely of CF isolates with the exception of one NCF isolate (BccBCC0048) belonging to cluster 10 from Germany. Genomes forming fastbaps clusters 4, 6, 12, and 13 belonged to clades III, VI, I, and VIII respectively, representing mixed isolating grouping (**Figure 1**). Correlation of country and clusters depicted in **Figure S3**.

**Figure 2:**
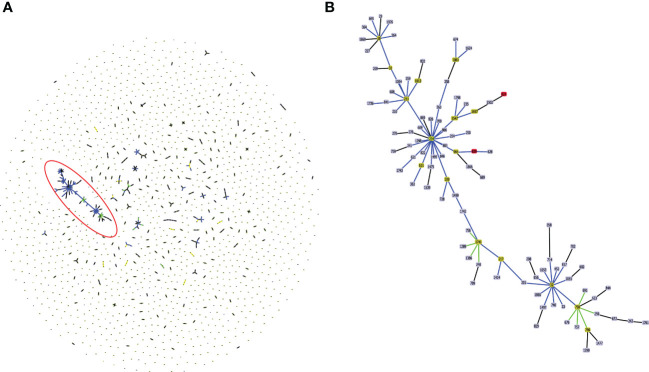
**(A)** Population structure analysis of available sequence types (ST) of *Burkholderia cepacia* complex in pubMLST database. The single ST represented by dot. Groups that are double locus variants linked by STs form clonal complex (CC). The largest CC31 is highlighted in the red eclipse. **(B)** Zoom image of the CC31, which is highlighted in **(A)**. Color code used by goeBURST tool indicates: Light blue, red node color - Common node, Dark Green node color-Sub-group founder, Light green node color- Group founder.

*In silico* MLST profiling categorized 245 CC31 isolates into 23 different STs. 43 isolates from our collection classified into ST807 (n=7), ST824 (n=5), ST839 (n=15), ST628 (n=4), ST208 (n=1), ST841 (n=1), ST826 (n=3), ST832 (n=2), ST822 (n=1), ST709 (n=1), ST217 (n=1), ST22 (n=1), and ST232 (n=1). Thirty-five isolates belonging to 11 different sequence types (except ST807 and ST22) were linked to CC31 (**Figure 2**). 5 STs (21.7%) were exclusively reported from India such as ST822, ST824, ST826, ST832, and ST841 (Gautam et al., 2016) indicating the genetic divergence of these isolates (**Figures 1**, **2**). ST241, ST628, and ST234 constitute both CF and NCF isolates. Pairwise SNP distance between NCF and CF isolates within ST628 and ST241 revealed fewer than eight differences suggesting a close relationship. SNP comparison of ST234 isolates revealed intercontinental transmission, where the difference of less than 23 SNP revealed the NCF isolate BccBCC0048 from Germany is closely related to 41 CF isolates from Canada than CF isolate BccAU1082 from the USA with 37 SNP differences. SNP analysis of ST832, ST826, ST824, and ST839 isolates from our collection suggest clonal nature with SNP difference ≤ 1 (**Table S5**). Correlation of STs and clusters depicted in **Figure S4**.

### Pan-genome analysis

**Figure 3:**
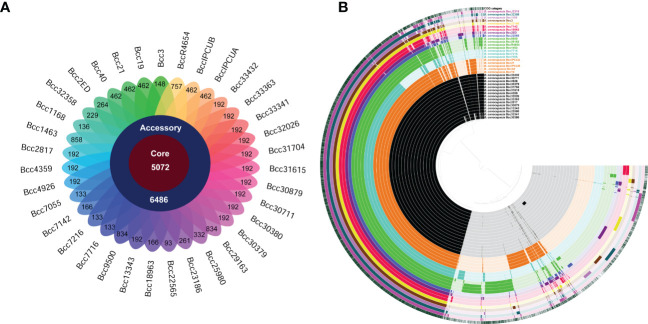
Distribution of gene cluster numbers in the pan-genome of CC31 isolates from India. **(A)** A flower plot schematic representation depicts the number of gene clusters (from inside to outside) in core, accessory, and unique genomes. **(B)** Comparative overview of pan- and core genomes obtained from the Anvi’o tool. The different *B cenocepacia* isolates grouped into sequence types (ST) are displayed (from inner to outer ring) by color black (ST839), orange (ST824), light blue (ST826), green (ST628), dark blue (ST208), purple (ST232), red (ST832), yellow (ST217), brown (ST841), light purple (ST822), and dark green (ST709). Last second ring represents reference *B cenocepacia* strain J2315. Last ring represents cluster of orthologous group categories. Each track indicates a genome, which is presented by number and color variations (dark/light), shows presence/absence of genes per genome.

We performed a pan-genome analysis of 35 *B. cenocepacia* isolates to ascertain the genomic repertoire of the CC31 population in India. The core and variable gene pool among the CC31 were revealed by pan-genome analysis. The core pan-plot clearly shows that with the inclusion of the new genomes, the pan-size rose exponentially, thus considered to have the “open” pan-genome (**Figure S5**). Pan-genome is classified into two categories: core genome and accessory genome. The core genome contained gene clusters in 100% of the genomes studied; the accessory genome included dispensable (genes shared by 2 to 35 isolates) and unique (genes present in a single isolate). A total of 11,558 gene clusters were found in the pan-genome of *B. cenocepacia* based on the comparison of the protein-coding genes. Compared to the gene clusters in the core genome (5,072 gene clusters, 43.8%), those in the accessory genome (6,486 gene cluster, 56.1%) contributed more to the pan-genome composition. The accessory genes were further divided into 2,035 gene clusters, disseminated solely among distinct isolates as a unique genome, and 4,451 gene clusters in the dispensable genome (**Figure 3A**). The pan and core genomes were displayed using the anvi’o tool. The protein sets present in each of the *B. cenocepacia* isolates enabling genome conservation and differentiation along with COG classification were also highlighted by the anvi’o display (**Figure 3B**). 7,601 (65.7%) of the 11,558 gene clusters in total were categorised into COG functional categories. The COG enrichment analysis showed that the COG class K (transcription, 545 gene clusters), class E (amino acid transport and metabolism, 492 gene clusters), class G (carbohydrate transport and metabolism, 332 gene clusters), class I (lipid transport and metabolism, 315 gene clusters), class M (cell wall/membrane/envelope biogenesis, 361 gene clusters), and class R (general function, 463 gene clusters) genes were more abundant in the gene clusters in the core genome of the isolates. In contrast, the genes for class C (energy production and conversion, 131 gene clusters), class L (replication, recombination, and repair, 154 gene clusters), class N (cell motility, 58 gene clusters), class P (inorganic ion transport and metabolism, 121 gene clusters), class T (signal transduction mechanisms, 105 gene clusters), class U (intracellular trafficking, secretion, and vesicular transport, 160 gene clusters), and class X (mobilomes, prophages, and transposons, 281 gene clusters) genes were highly abundant in the accessory genome. Transcription (Class K, 106 gene clusters); replication, recombination, and repair (Class L, 95 gene clusters); and mobilomes, prophages, and transposons (class X, 87 gene clusters) are abundant classes in singletons, indicating the tendency of genomes to acquire horizontally acquired elements for adaptation to diverse niches. Core and accessory gene clusters are summarized in **Table S6**.

### Resistance gene distribution

**Figure 4:**
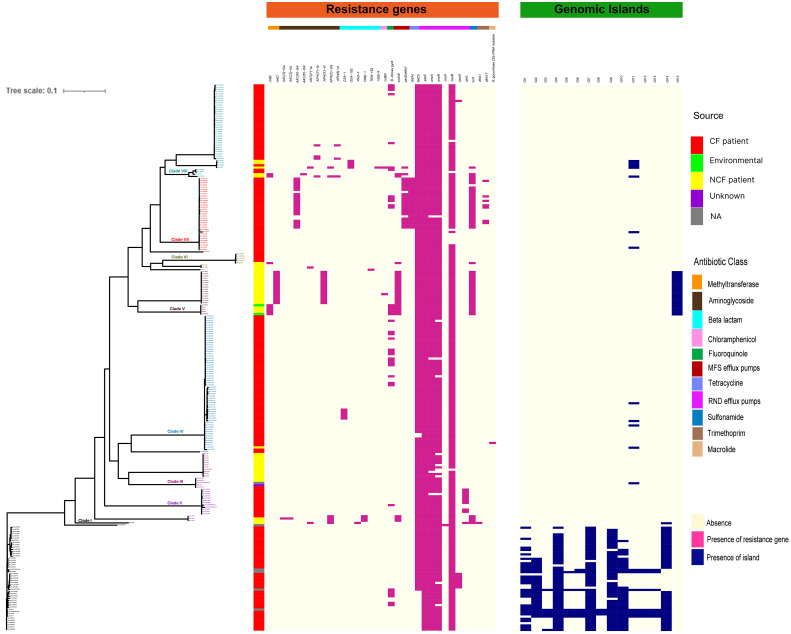
Distributions of mutations, resistance genes, and genomic islands in the CC31 lineage. Pink squares represent presence of resistance genes while blue squares represent the presence of islands.

Considering the clinical significance of antimicrobial resistance (AMR) in *B. cenocepacia*, we compared the resistance genes in 245 CC31 isolates from different clades. In total 34 resistance genes concordant with different antibiotic classes (β-lactams, aminoglycoside, chloramphenicol, fluoroquinolone, tetracycline, sulphonamide, trimethoprim, and macrolide) were present in isolates from the eight clades. Resistance to five genes belonging to antibiotic class tetracycline and resistance-nodulation-cell division antibiotic efflux pump were present in nearly all the isolates. In every clade, CF isolates had a higher number of AMR genes than NCF isolates, indicating a high degree of resistance in these isolates (**Figure 4**). Mutation in *gyrA* gene encoding resistance to fluoroquinolones was present in 33 isolates (16.7%, 33/197) from CF patients and 5 ST824 NCF isolates (11.3%, 5/44). ST824 isolates were outbreak isolates from the neonatal ward in India known to have caused devastating effects (Patil et al., 2017). 23S rRNA mutation conferring resistance to azithromycin was present in a single isolate VC14761 from a CF patient in Canada. 61.7% (21/34) isolates from Clade VII had significantly more AMR genes belonging to 7 different classes considered multidrug resistant. Isolates from Clade V and VII were resistant to sulfonamides. Two CF isolates (BccVC6905, BccVC2307) and one NCF isolate (Bcc32358) harbored gene *APH(3’’)-Ib* for streptomycin antibiotic resistance. NCF isolates from India contain one or more resistant genes for tetracycline, fluoroquinolone, chloramphenicol, and aminoglycoside antibiotics. ST628 isolates contain beta-lactamase gene *OXA-192*, whereas isolate Bcc2ED, Bcc3, and Bcc7055 carry *PAU-1*, *TEM162*, and *PME-1* genes, respectively. ST839 isolates were also resistant to amikacin antibiotics. Apart from antibiotic-resistant genes, isolates Bcc1463, Bcc23186, and Bcc2ED also encode genes *qacEdelta1* and *qacL*, providing resistance to disinfecting agents and antiseptics. NCF isolate Bcc1463 is an extreme drug resistant strain with resistance to chloramphenicol (*catB3*) and beta-lactamase containing antibiotics (*OXA192*, *VEB9*), along with resistance to other major antibiotic classes. Methyltransferase genes (*rmtB* and *rmtD*) encoding resistance to aminoglycosides were reported in ST824 and ST839 NCF isolates, respectively. The comparison of the resistance profile of the NCF isolates with that of CF isolates has been described in **Table S7**.

### Antimicrobial susceptibility profiling

*In vitro* antimicrobial susceptibility testing (AST) of 35 isolates from India was performed against the recommended antibiotics and tetracycline to correlate with the information of resistance genes available on sequencing (**Table 1**). Majority of isolates were resistant to chloramphenicol (77%, 27/35) followed by levofloxacin (34%, 12/35) antibiotic. ST826 and ST824 isolates were resistant to levofloxacin and chloramphenicol, whereas ST839 isolates were resistant to chloramphenicol. Isolates Bcc1168 and Bcc31704 (5.7%, 2/35) were resistant to ceftazidime antibiotic, whereas isolates Bcc2ED, Bcc32358, Bcc1463, Bcc29163, Bcc33363, and Bcc31704 (17%, 6/35) were resistant to cotrimoxazole. Isolates Bcc22565, Bcc1168, Bcc31704 (8.5%, 3/35) were resistant to antibiotic meropenem. All isolates were susceptible to piperacillin-tazobactam except isolate Bcc18963 (2.8%, 1/35). Isolates are resistant to first- and second-line agents, such as ceftazidime, meropenem, and penicillins (mainly piperacillin), so there is a need to switch to other alternative methods to combat *B*. *cenocepacia* infections. Resistance to clinically significant antibiotics, chloramphenicol, levofloxacin, and tetracycline correlates with the presence of RND efflux pumps (Podnecky et al., 2015) and tetracycline resistance gene in these isolates, which is in accordance with the AST profile, indicating genome sequencing as a probable tool for clinicians to diagnose and treat complicated *B. cenocepacia* infections.

**Table 1:** Antimicrobial susceptibility testing of 35 CC31 isolates from India.

Isolate ID	CAZ	COT	MEM	MIN	LEV	CHL	TET	PTZ
**Bcc_7216**	S	S	S	S	R	R	R	S
**Bcc_7716**	S	S	S	S	R	R	S	S
**Bcc_7055**	S	S	S	S	R	R	S	S
**Bcc_2ED**	S	R	S	S	S	S	R	S
**Bcc_7142**	S	S	S	S	S	R	S	S
**Bcc_18963**	S	S	S	S	R	S	S	R
**Bcc_1168**	R	S	R	S	S	S	S	S
**Bcc_32358**	S	R	S	S	R	R	R	S
**Bcc_23186**	S	S	S	S	S	R	R	S
**Bcc_1463**	S	R	S	S	R	S	R	S
**Bcc_R4654**	S	S	S	S	S	S	S	S
**Bcc_9500**	S	S	S	S	S	S	S	S
**Bcc_29163**	S	R	S	S	R	S	R	S
**Bcc_3**	S	S	S	S	S	S	R	S
**Bcc_IPCUB**	S	S	S	S	R	R	R	S
**Bcc_IPCUA**	S	S	S	S	R	R	R	S
**Bcc_21**	S	S	S	S	R	R	R	S
**Bcc_40**	S	S	S	S	R	R	R	S
**Bcc_19**	S	S	S	S	R	R	R	S
**Bcc_4359**	S	S	S	I	S	R	R	S
**Bcc_33432**	S	S	S	S	S	R	R	S
**Bcc_33363**	S	R	S	I	S	R	R	S
**Bcc_33341**	S	S	S	I	S	R	R	S
**Bcc_32026**	S	S	S	I	S	R	R	S
**Bcc_31704**	R	R	R	I	S	R	R	S
**Bcc_31615**	S	S	S	I	S	R	R	S
**Bcc_30879**	S	S	S	I	S	R	R	S
**Bcc_30711**	S	S	S	S	S	R	R	S
**Bcc_30380**	S	S	S	I	S	R	R	S
**Bcc_30379**	S	S	S	S	S	R	R	S
**Bcc_2817**	S	S	S	S	S	R	R	S
**Bcc_25980**	S	S	S	I	S	R	R	S
**Bcc_4926**	S	S	S	S	S	R	R	S
**Bcc_13343**	S	S	S	I	S	R	R	S
**Bcc_22565**	S	S	R	S	S	R	R	S

R, resistant; S, susceptible; I, intermediate; CAZ, Ceftazidime; MEM, Meropenem; COT, Cotrimoxazole; LEV, Levofloxacin; MIN, minocycline; TET, Tetracycline; CHL, Chloramphenicol; PTZ, Piperacillin-tazobactam.

### Virulence gene distribution

**Figure 5:**
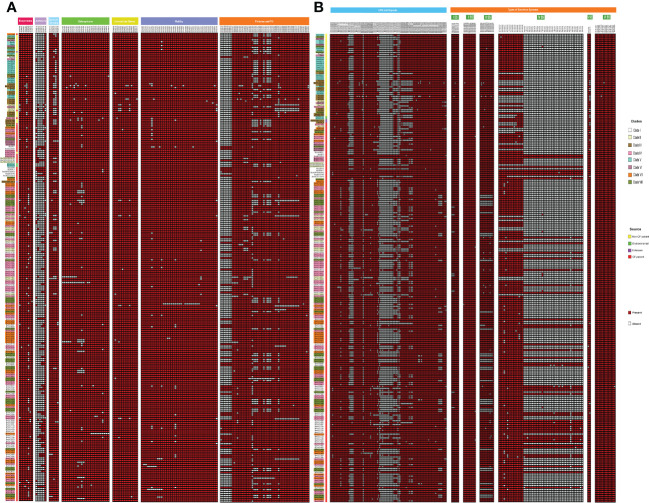
Heatmap depicting the virulence profile of 245 CC31 isolates. Red square box represents presence of virulence genes belonging to different categories. **(A)** Exoproteins, Adhseins, Quorum sensing, Siderophores, Motility, Fimbriae and pili. **(B)** LPS capsule, other capsules, and secretion systems.

We further performed a comparative analysis of 352 known virulence genes in *B. cenocepacia* to estimate the pathogenic potential of 245 CC31 isolates. A series of genes associated with zinc metalloproteases, exoproteins, lipopolysaccharide (LPS) biosynthetic clusters, adhesion, fimbriae, flp-type pilli, type IVa pilus, iron uptake, quorum sensing, motility, intracellular stress, and secretion systems Type (I, II, V, VI) were widely distributed among isolates of each clade. The major genes involved in the iron uptake like *fhuB*, *fhuF*, *orbS*, *orbI*, *orbJ*, *pvdA*, *orbA*, *fptA*, and *phuV* are present in all the 245 CC31 isolates. The N-acylhomoserine lactone quorum-sensing regulator involved in the regulation of virulence genes such as *cepIR* and *cciIR* (Eberl, 2006) is present in all the isolates. Exopolysaccharide also contributes to virulence in Bcc, whose expression is strain specific, and its expression increases the virulence (Chung et al., 2003). The genes for exoprotein production like metalloproteases *ZmpA* and *ZmpB* are present among all the isolates, but phosphatidylinositol-specific Phospholipase C is absent in 41 (20.8%, 41/197) CF isolates. The flagellum makes the bacteria motile and provides adhesion that helps invade the host cell serving as an important virulence factor (Tomich et al., 2002). Motility involves chemotaxis genes (*cheABRWYZ*), peritrichous flagellar genes (*flg*, *fli*, and *flh*), and flagellar motor genes (*motA* and *motB*) were distributed in the majority of the CC31 isolates. Adhesion genes (cable pili *cblA*, *adhA*) play a vital role in invading the respiratory epithelium (Urban et al., 2005). Interestingly, *cblA* gene responsible for the production of cable pili (Sun et al., 1995) along with *cblS* and *cblT*, sensor histidine kinases, is absent from all NCF isolates (97%, 43/44) except in Bcc2ED. Cable pilus-associated adhesin, *adhA* is also absent in NCF isolates (86.3%, 38/44) except in six isolates BccAU1098, Bcc28_4, BccAU11115, BccBCC0048, Bcc3 and Bcc30379. Interestingly, *cblA* and *adhA* are present in all the CF isolates of *B. cenocepacia*. Among secretion systems, type III is absent from approximately 29 CF isolates (14.7%, 29/197) and type IV is absent in 6 NCF (13.6%, 6/44) and 18 CF isolates (9.1%, 18/197). Type IV secretion system on the conjugative plasmid is exclusively present in CF isolates (n=197, 100%) (**Figure 5**). The comparison of the virulence profile of the NCF isolates with that of CF isolates has been described in **Table S8**.

### Genomic islands and phages

**Figure 6:**
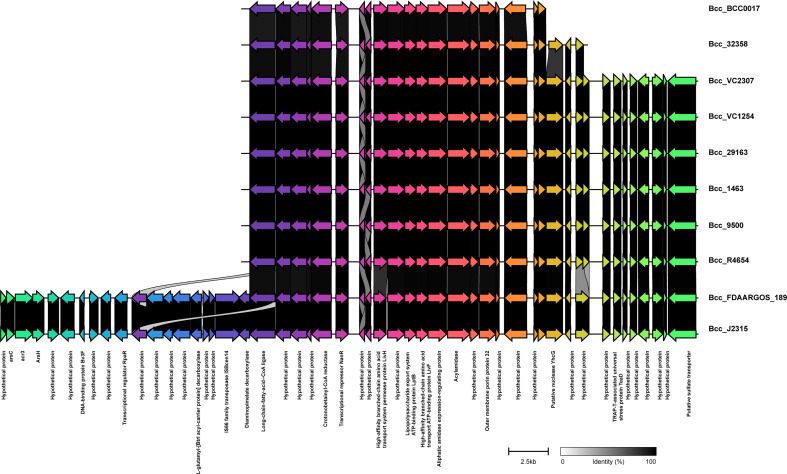
Comparison of pathogenicity island GI11 between CC31 isolates across clades. Genes are represented by arrowed boxes with labels. Percent identity is represented by connecting links between genes.

We next studied the distribution of genomic islands (GI) in the CC31 lineage because they are a significant component of the Bcc genome that might play an important role in adaptation to diverse niches and evolution of virulence (Dobrindt et al., 2004). The complete genome sequence of *B. cenocepacia* J2315 (reference strain) revealed that it contains fourteen genomic islands, namely GI1 to GI14, occupying 9.3% of the 8.06 Mb genome (Holden et al., 2009). Our previous study discovered a novel genomic island named GI15 in *B. cenocepacia* isolates from a nosocomial outbreak in India (Patil et al., 2017). A BLAST search against nucleotide sequences of genomic islands revealed that several genomic islands (GI1, GI2, GI4, GI7, GI9, GI10, GI14) are present in the majority of clade I isolates (**Figure 4**). The GI11 is one of the well-characterized pathogenicity islands that encodes *esmR*, called BCESM, and is present in isolates across every clade. This island is 44.1kb reported to be located on chromosome two and contained forty-three coding sequences (Baldwin et al., 2004). The genes for intracellular stress (*TeaD*), putative sulfate transporter, and a quorum-sensing system (*rpaR*) are present on this island, which play an important role in virulence, resistance, and inflammatory process (Van Acker et al., 2014). Comparison of GI11 across isolates revealed that arsenic resistance genes (*arsC*, *acr3*, *arsH*) and IS66 family transposase ISBcen14 were exclusively present in reference strain BccJ2315 and CF isolate BccFDAARGOS189 from the USA. The ST628 and ST709 isolates from the Indian Bcc population differ in GI11 and genes encoding the transposase ISBcen14, the transcriptional repressor *rpaR*, and the arsenic resistance have been lost owing to their adaptation. In addition, ST709 isolates lost genes encoding putative sulfate transporter (**Figure 6**). GI15 is 107 kb island and carries genes involved in pathogenesis including well-characterized auto-transporter adhesion (Patil et al., 2017). GI15 is exclusively present in all clade V isolates. GI15 is absent in reference isolate *B. cenocepacia* J2315 and other global CC31 isolates. GI15 is exclusively present in ST824 (Patil et al., 2017) and ST839 (Saroha et al., 2022) isolates. The isolates are collected from two different states of India. The comparison of the genomic islands of the NCF isolates with CF isolates has been described in **Table S9**.

**Figure 7:**
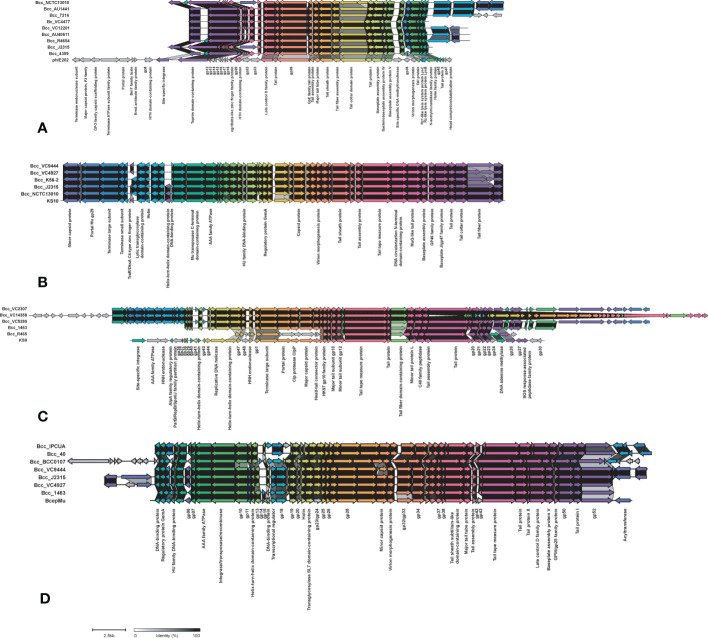
Comparison of phages phiE202 **(A)**, KS10 **(B)**, KS9 **(C)**, and BcepMu **(D)** between CC31 isolates across clades. The colored arrowed boxes and labels below boxes represent genes and gene product respectively. Connecting links between genes represent percent identity. The unlabeled arrows represent hypothetical genes.

Phages carry toxin genes and enzymes, enabling their host to infect other organisms by overcoming their defense system and eventually contribute to host pathogenicity. We compared Bcc phages in the NCBI database in the CC31 lineage (Roszniowski et al., 2018). Out of 39 phages, 13 phages were present in the CC31 lineage. Phage phiE202 (58/245), KS10 (45/245), KS9 (38/245), BcepMu (33/245), and KL3 (28/245) are predominantly found in CC31 lineage, whereas phiE255 (17/245), phi1026b (11/245), ST79 (3/245), BcepNY3 (3/245), phiE125 (2/245), Bcep1 (2/245), BcepC6B (2/245), and vB_BceM_AP3 (1/245) were present in few isolates. Phages phiE202 (16/35), KL3 (11/35), BcepMu (6/35), BcepC6B (6/35), KS9 (4/35), phiE255 (2/35), Bcep1 (2/35), and BcepNY3 (1/35) were found in Indian CC31 population. The variation in major phages is depicted in **Figure 7** between representative isolates in each clade. Phage phiE202 belongs to the Myoviridae family and is a phage of *B. thaliandensis*, whereas phages BcepMu and KS10 are phages of *B. cenocepacia*, and KL3 is phage of *B. amfibaria*. KS9 belongs to Siphoviridae and is a phage of *B. pyrrocinia* (Roszniowski et al., 2018). Apart from basic phage machinery, phages carry additional genes conferring special advantages to their host. phiE202 carry genes encoding antimicrobial enzyme N-acetylmuramidase, peptidoglycan hydrolase (*LysA*), mycolylarabinogalactan esterase (*LysB*), and holin, which work together in the lysis of host cell membrane were present in isolates from every clade except clade III and IV. In addition, phiE202 contains a toxin-antitoxin (*BrnT*, *BrnA*) system, which is absent from these isolates (**Figure 7A**). All genes of phage KS10 and BcepMu (except gene acyltransferase) were present in isolates. Phage KS10 is exclusively present in CF isolates (**Figure 7B**). phiE255 carry genes encoding minor capsid protein gp7, baseplate central spike complex protein gp27, terminase gp28, formyl transferase gp30, and non-glycosylated membrane associated protein gp31 which were absent from isolates. Phage phiE255 was detected in Bcc22565, Bcc2ED (NCF) and Bcc NCTC13010, BccBCC0138 (CF) isolates (**Figure S6B**). Phage ST79 belongs to the Myoviridae family and is a lytic phage of *B. pseudomallai*. It is exclusively present in Indian NCF isolates Bcc1463, Bcc29163, and CF isolate BccR4654. It carries type II HicA toxin-HicB antitoxin system and IS21 family transposase (**Figure S6C**). A Comparison of minor phages is depicted in supplementary figure **Figure S6**. The phages profile in 245 CC31 isolates has been described in **Table S10**.

## Discussion

CC31 lineage is the largest clonal complex in the *Burkholderia cepacia* complex, with *Burkholderia cenocepacia* being the predominant species (Gautam et al., 2016). The majority of *B. cenocepacia* isolates reported to date are isolated from CF patients. Very few of them have been reported from other nosocomial sources. Several studies have described CC31 isolates causing havoc in hospital settings globally because they are intrinsically resistant to major antibiotics and disinfecting agents (Nunvar et al., 2017). Bacteria involve various mechanisms of antibiotic resistance such as alteration in cell permeability, antibiotic target modifying or degrading enzymes, and limited access to antibiotics (Blair et al., 2015). A significant understanding of CC31 lineage is required to develop novel therapeutics to treat *B. cenocepacia* infections. Whole genome-based resistome analysis of 245 CC31 isolates revealed that CF isolates possess a high number of resistance genes. Among 245 isolates of the CC31 lineage studied here, we found 34 resistance genes amongst different antibiotic classes. Out of 34 AMR genes analyzed, five genes were present in nearly all the isolates constituting core resistance genes, implicating their role in intrinsic resistance to aminoglycosides, tetracyclines, and fluoroquinolones. Additionally, resistome comprises of efflux pumps and regulators, which assist other factors or genes in antibiotic resistance. Apart from core resistance genes, CF isolates were resistant to trimethoprim, cephalosporins, gentamicin, macrolides, and disinfecting agents, whereas NCF isolates were resistant to phenicols and carbapenems. Since trimethoprim and sulfonamide antibiotics are used frequently in treating Bcc infections in CF patients (Gautam et al., 2015), the analysis in the present study underlined the genes involved in resistance to these antibiotics, thus there is a dire necessity to perform *in vitro* studies and switch to alternative options.

Virulence is an essential determinant of bacterial pathogenicity (Drevinek and Mahenthiralingam, 2010). Of 352 virulence genes analysed in CC31 isolates, 317 genes constitute core virulome, indicating that they might have contributed to adaptation and evolution of virulence in the CC31 lineage. Core virulence genes belong to zinc metalloproteases, surface polysaccharides (exoproteins and LPS biosynthetic clusters), adhesion, flagella, iron uptake, quorum sensing, motility, intracellular stress, and secretion systems. Iron is an essential element for the survival of bacterial pathogens inside the host during infection as they face a shortage of free iron due to the binding of this metal ion to transferrin, ferritin, and lactoferrin. Thus, iron acquisition genes are not virulence factors, but the systems used to sequester iron from an iron-depleted environment within the host ultimately play a role in virulence (Visser et al., 2004). To fulfil the iron requirements, members of Bcc produce different iron-chelating compounds known as siderophores like ornibactin, salicylic acid, phyochelin, cepabactin, and cepaciachelin (Thomas, 2007). *B. cenocepacia* majorly produces primary ornibactin and secondary pyochelin siderophores. Although NCF isolates have a comparable number of virulence genes as CF isolates, they lack conjugative plasmid encoding type IV secretion system and cable pilus-associated adhesion genes.

Genomic islands are large genomic regions (more than 10kb in size) flanked by tRNA, and direct repeats carry genes involved in host adaptation and evolution. A subset of the genomic island called pathogenicity islands carries numerous virulence-associated genes enabling bacterial pathogenicity and adaptive evolution through horizontal transfer events (Dobrindt et al., 2004). There are 15 genomic islands reported in Bcc. GI11 and GI15 are pathogenicity-associated islands (Holden et al., 2009; Patil et al., 2017). A comparison of genomic islands revealed that the majority of GIs are present in clade I isolates. There is variation in GI11 across isolates from each clade. GI15 is exclusively present in ST824 and ST839 isolates from India, possibly owing to its mobility and existence in India with the extreme burden of drugs and antibiotics in hospital settings.

Bacteriophages have tremendous impact on the proliferation and evolution of pathogenic bacteria. They carry numerous genes which can contribute to bacterial virulence. After entering the bacterial cell, they can either follow a lysogenic or lytic life cycle. Temperate phages integrate into the host chromosome and follow a lysogenic cycle. They are characterized by the presence of integrase or recombinase (Borodovich et al., 2022). Lysogenic phages a play role in the transmission of antibiotic resistance in the *Streptococcus* and *Enterobacteriaceae* family (Ubukata et al., 1975; Colavecchio et al., 2017). We analyzed 39 phages, out of which 13 phages were present in CC31 isolates. All phages were temperate phages except for one lytic phage, ST79. Lytic phage ST79 is exclusively found in Indian isolates owing to the horizontal acquisition of phage from different pathogenic species *Burkholderia pseudomallai* within the *Burkholderia* genus.

There are a few limitations of this study. Firstly, the number of NCF isolates sampled needed to be larger to make conclusive remarks. Secondly, there is a lack of detailed epidemiological information, which may have affected the epidemiological analysis of CC31 lineage. Further experimental evidence is required to prove the similar virulence potential of CF and NCF isolates.

## Conclusion

The present study provides first and considerate insight into the epidemiological transmission, evolutionary relationship, pan-genome analysis, virulence, antibiotic resistance, and mobile genetic elements of the CC31 lineage from India. Global phylogenetic analysis indicate that NCF isolates have evolved independently of CF isolates. NCF isolates are resistant to first-and-second line of antibiotics along with disinfecting agents and antiseptics because of heavy antimicrobial usage in hospital settings in India. Isolate Bcc1463 is an extreme drug resistant strain, with the potential to transfer resistance to other strains thus further worsening the AMR situation. In addition, NCF isolates have a comparable number of virulent genes to CF isolates. Experimental validation and more data from other sources are required to predict the epidemic and virulence potential of the CC31 lineage. Additionally, there is a need for monitoring the use of antimicrobials and other disinfectants for environmental hygiene, as well as rapid genome-based investigation of multidrug resistant strains in nosocomial infections.

## Data availability statement

The datasets presented in this study can be found in online repositories. The names of the repository/repositories and accession number(s) can be found in the article/**Supplementary Material**.

## Ethics statement

Ethical approval for this study was submitted and approved by the Institutional Biosafety Committee, Institute of Microbial Technology (IMTECH) Chandigarh. Informed consent was not needed since this project was a retrospective study and all bacterial samples from patients were for scientific use.

## Author contributions

TS and PPP involved in identification and assembly of the isolates. TS involved in data analysis and drafted manuscript-taking inputs from all authors. RR carried out cluster comparison visualization by taking inputs from TS. SK involved in genome assembly of some isolates. RK and LS involved in isolation of strains and carried out antimicrobial susceptibility testing. TS, PPP, VG, and PBP participated in design of the study. PBP, LS, and VG coordinated the study and applied for the funding. All authors contributed to the article and approved the submitted version.

## Supplementary material

Supplementary Figure 1 Phylogenomics and taxonogenomic assessment of species of clinical *Burkholderia cepacia* complex isolates. **(A)** Heat map of ANI values among Bcc clinical isolates with type strain of *Burkholderia* genus. **(B)** Maximum-likelihood phylogeny of catenated core gene sequences from type strains of *Burkholderia* genus.

Supplementary Figure 2 Phylogenetic structure of 245 CC31 isolates. Clades I-VIII are marked by background color fill. Data on the source of isolation and fastbaps clusters are depicted on the tree (from inner to outer circle).

Supplementary Figure 3 Phylogenetic structure of 245 CC31 isolates. Clades I-VIII are marked by background color fill. Data on the country of isolation and fastbaps clusters are depicted on the tree (from inner to outer circle).

Supplementary Figure 4 Phylogenetic structure of 245 CC31 isolates. Clades I-VIII are marked by background color fill. Data on the MLST classification and fastbaps clusters are depicted on the tree (from inner to outer circle).

Supplementary Figure 5 Pan-genome curve of *B*. *cenocepacia* CC31 isolates.

Supplementary Figure 6 Comparison of minor phages KL3 **(A)**, phiE255 **(B)**, ST79 **(C)**, and vB_BceM_AP3 **(D)** between CC31 isolates across clades. The colored arrowed boxes and labels below boxes represent genes and gene product respectively. Connecting links between genes represent percent identity. The unlabeled arrows represent hypothetical genes.
