# Supplementary material for: Genomic features, antimicrobial susceptibility, and epidemiological insights into Burkholderia cenocepacia clonal complex 31 isolates from bloodstream infections in India
Source: Front Cell Infect Microbiol. 2023 Apr 19;13:1151594. doi: 10.3389/fcimb.2023.1151594 (PMC10155701; doi:10.3389/fcimb.2023.1151594)
Supplement: Supplementary file 1 [file DataSheet_1.pdf]

Table S1: Assembly statistics of 43 *Burkholderia cenocepacia* isolates

Isolate ID	Average Genome coverage	Contig Count	GC%	N50 (bp)	Genome Size (bp)	Completeness	Contamination
Bcc_32358	167.45	195	67.24	191419	7948054	99.87	0.46
Bcc_2ED	182.75	177	66.85	182134	8385097	99.87	1.44
Bcc_31704	106.16	198	66.78	154754	8276588	99.66	0.68
Bcc_30879	158.91	183	66.78	178277	8281799	99.66	0.54
Bcc_5766	183.41	185	67.12	201001	8053757	99.87	1.18
Bcc_5771	190.64	155	67.13	219442	8055873	99.87	0.71
Bcc_5218	154.63	174	67.13	231875	8052221	99.87	0.77
Bcc_32185	213.93	234	67.00	200059	8247772	99.87	1.07
Bcc_R7748	213.38	188	67.00	219441	8244312	99.87	0.71
Bcc_R4654	167.02	223	66.79	144878	8567995	99.87	0.26
Bcc_29163	157.26	236	66.73	161181	8641890	99.87	0.20
Bcc_1125	118	123	66.99	219040	8262210	99.46	0.71
Bcc_R7746	185.95	161	67.03	219441	8228682	99.87	0.71
Bcc_3	105.23	216	67.13	204748	7920062	99.87	1.29
Bcc_1168	85.485	97	67.52	231969	7470410	99.87	0.03
Bcc_21	102.79	231	66.39	168721	8698170	99.87	1.20
Bcc_19	84.36	242	66.40	163818	8695676	99.87	1.41
Bcc_7055	80.09	111	67.31	219170	6693300	99.87	0.25
Bcc_7216	138.27	114	67.35	246798	6651036	99.87	1.16
Bcc_7716	104.18	91	67.35	379694	6650446	99.87	0.25

Bcc_IPCUA	95.85	229	66.39	154158	8695683	99.87	1.29
Bcc_9500	65.44	293	66.71	81638	8670523	99.83	0.50
Bcc_22565	85.97	127	67.11	161308	8036403	99.87	0.12
Bcc_25980	78.55	163	66.29	143744	8444026	99.66	0.54
Bcc_30379	114.16	275	66.73	157316	8312165	99.66	0.63
Bcc_30380	47.44	178	66.92	119895	8057648	99.66	0.54
Bcc_IPCUB	203.01	248	66.40	176643	8677073	99.87	1.42
Bcc_7142	96.69	307	67.36	158740	7703347	99.87	0.44
Bcc_33363	146.34	187	66.78	154753	8277548	99.66	0.87
Bcc_33432	158.03	180	66.78	151102	8275714	99.66	0.54
Bcc_33341	166.14	162	66.93	159908	8039403	99.66	0.54
Bcc_2817	158.07	164	66.78	154720	8272676	99.66	0.54
Bcc_4359	155.72	176	66.78	151102	8275364	99.66	0.55
Bcc_4926	181.52	160	66.78	160919	8278734	99.66	0.54
Bcc_31615	187.14	153	66.78	173481	8278801	99.66	0.54
Bcc_13343	138.77	155	66.79	147837	8279040	99.66	0.54
Bcc_30711	181.75	218	66.78	151187	8281111	99.66	0.74
Bcc_32026	176.69	199	66.78	151102	8279710	99.66	0.54
Bcc_18963	172.42	203	67.38	192991	7684429	99.87	0.15
Bcc_23186	180.87	211	67.19	169439	8015634	99.87	0.12
Bcc_1810	214.28	221	67.05	182410	8177168	99.46	0.13
Bcc_1463	199.69	237	66.99	160924	8344115	99.87	0.25
Bcc_40	99.36	247	66.39	163818	8730110	99.87	1.43
