# Supplementary material for: Genomic features, antimicrobial susceptibility, and epidemiological insights into Burkholderia cenocepacia clonal complex 31 isolates from bloodstream infections in India
Source: Front Cell Infect Microbiol. 2023 Apr 19;13:1151594. doi: 10.3389/fcimb.2023.1151594 (PMC10155701; doi:10.3389/fcimb.2023.1151594)
Supplement: Supplementary file 2 [file DataSheet_2.pdf]

Table S2: Genome characteristics of 43 *Burkholderia cenocepacia* isolates

**Isolate ID**	**Isolation Source**	**CDS**	**No. of tRNAs**	**No. of rRNAs**	**ST**	**BioProject No.**	**Accession No.**
Bcc_32358	Blood	7345	58	8	709	PRJNA756906	JAIMHU000000000
Bcc_2ED	Blood	7759	59	9	232	PRJNA756946	JAIMII000000000
Bcc_31704	Blood	7715	57	6	839	PRJNA756906	JAIMHF000000000
Bcc_30879	Blood	7692	58	7	839	PRJNA756906	JAIMHL000000000
Bcc_5766	Blood	7420	60	6	807	PRJNA756906	JAIMHA000000000
Bcc_5771	Blood	7400	60	5	807	PRJNA756906	JAIMHZ000000000
Bcc_5218	Blood	7417	60	5	807	PRJNA756947	JAIMIJ000000000
Bcc_32185	Blood	7675	61	8	807	PRJNA756906	JAIMHB000000000
Bcc_R7748	Respiratory	7632	60	7	807	PRJNA756906	JAIMHW000000000
Bcc_R4654	Respiratory	8007	60	6	628	PRJNA756906	JAIMHP000000000
Bcc_29163	Blood	8096	58	8	628	PRJNA756906	JAIMHQ000000000
Bcc_1125	Blood	7588	62	14	807	PRJNA381553	NBWJ00000000
Bcc_R7746	Respiratory	7604	61	8	807	PRJNA756906	JAIMHX000000000
Bcc_3	Blood	7298	62	15	841	PRJNA275518	JYMW00000000
Bcc_1168	Respiratory	6758	61	10	822	PRJNA381610	NBWN00000000
Bcc_21	Blood	8132	63	17	824	PRJNA275520	JYMY00000000
Bcc_19	Blood	8140	63	18	824	PRJNA275519	JYMX00000000
Bcc_7055	Blood	6063	62	17	826	PRJNA381544	NBWG00000000
Bcc_7216	Blood	6018	60	15	826	PRJNA381551	NBWI00000000
Bcc_7716	Respiratory	6004	60	18	826	PRJNA381550	NBWH00000000

Bcc_IPCUA	Amikacin vial	8138	65	18	824	PRJNA275524	JYNB00000000
Bcc_9500	Blood	8185	61	16	628	PRJNA381528	NBWK00000000
Bcc_22565	Blood	7399	62	13	208	PRJNA381603	NBWL00000000
Bcc_25980	Blood	7883	61	11	839	PRJNA381529	NBWD00000000
Bcc_30379	Blood	7814	62	16	839	PRJNA381538	NBWF00000000
Bcc_30380	Blood	7449	59	14	839	PRJNA381531	NBWE00000000
Bcc_IPCUB	Amikacin vial	8141	56	10	824	PRJNA275525	JYNC00000000
Bcc_7142	Blood	7126	61	14	832	PRJNA381605	NBWM00000000
Bcc_33363	Blood	7698	57	7	839	PRJNA756906	JAIMHM000000000
Bcc_33432	Blood	7680	58	11	839	PRJNA756906	JAIMHD000000000
Bcc_33341	Blood	7424	56	8	839	PRJNA756906	JAIMHC000000000
Bcc_2817	Blood	7678	57	9	839	PRJNA756906	JAIMHN000000000
Bcc_4359	Blood	7692	57	7	839	PRJNA756906	JAIMHH000000000
Bcc_4926	Blood	7667	60	6	839	PRJNA756906	JAIMHK000000000
Bcc_31615	Blood	7673	58	7	839	PRJNA756906	JAIMHI000000000
Bcc_13343	Blood	7670	57	7	839	PRJNA756906	JAIMHJ000000000
Bcc_30711	Blood	7723	57	6	839	PRJNA756906	JAIMHG000000000
Bcc_32026	Blood	7706	57	8	839	PRJNA756906	JAIMHE000000000
Bcc_18963	Blood	6998	58	6	832	PRJNA756906	JAIMHV000000000
Bcc_23186	Blood	7394	56	5	217	PRJNA756906	JAIMHT000000000
Bcc_1810	Blood	7647	59	10	22	PRJNA756906	JAIMHS000000000
Bcc_40	Blood	8180	63	18	824	PRJNA275521	JYMZ00000000
Bcc_1463	Blood	7751	57	8	628	PRJNA756906	JAIMHR000000000
