# Supplementary material for: Genomic features, antimicrobial susceptibility, and epidemiological insights into Burkholderia cenocepacia clonal complex 31 isolates from bloodstream infections in India
Source: Front Cell Infect Microbiol. 2023 Apr 19;13:1151594. doi: 10.3389/fcimb.2023.1151594 (PMC10155701; doi:10.3389/fcimb.2023.1151594)
Supplement: Supplementary file 3 [file DataSheet_3.pdf]

Table S4: Number of isolates from different isolation source

**Source**	**CF**	**NCF**	**Environmental**	**Unknown**
Total No. of isolates	197	44	2	2
