# Supplementary figures and images for: Genomic features, antimicrobial susceptibility, and epidemiological insights into Burkholderia cenocepacia clonal complex 31 isolates from bloodstream infections in India
Source: Front Cell Infect Microbiol. 2023 Apr 19;13:1151594. doi: 10.3389/fcimb.2023.1151594 (PMC10155701; doi:10.3389/fcimb.2023.1151594)

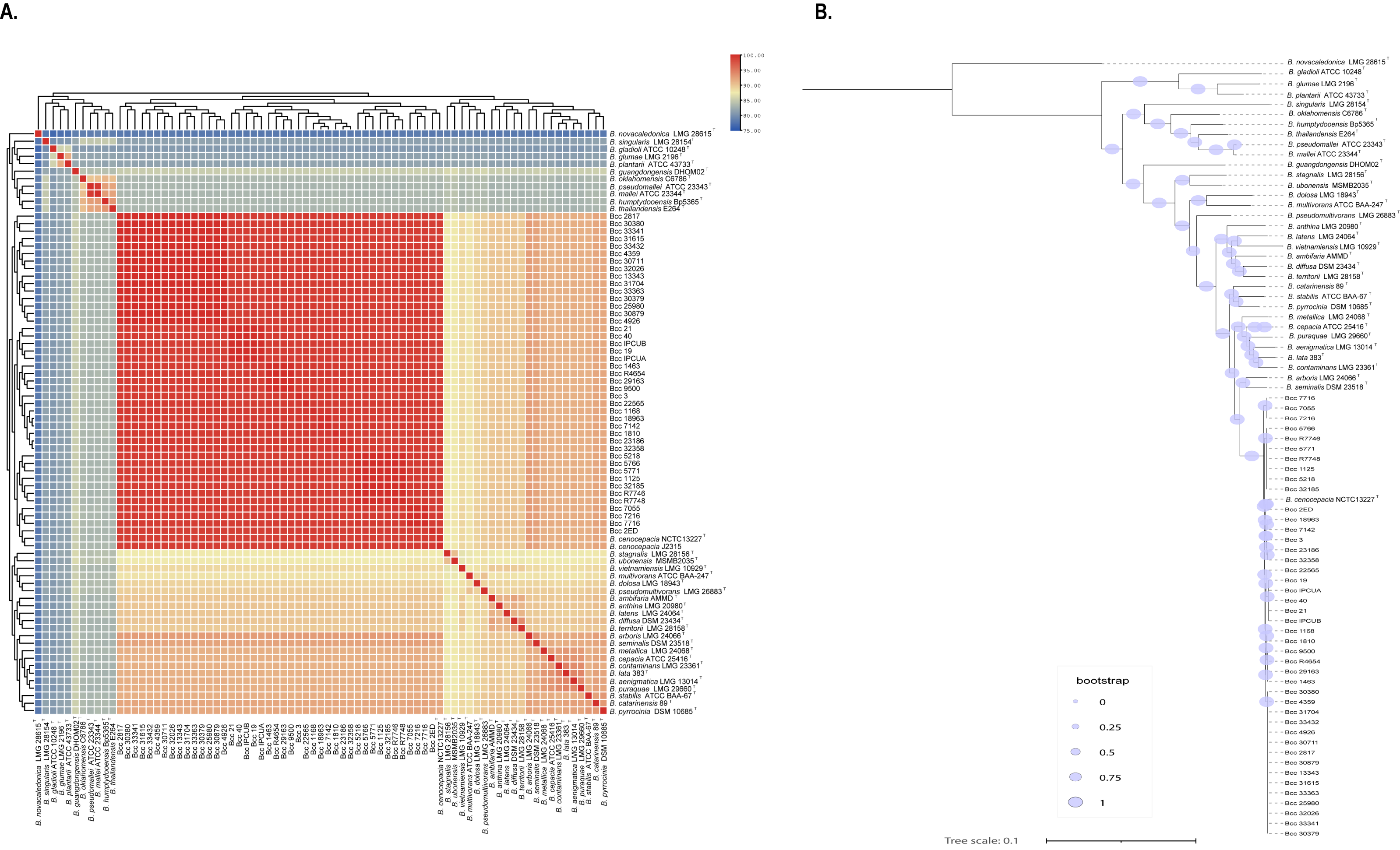

Supplement: Supplementary Figure 1 — Phylogenomics and taxonogenomic assessment of species of clinical Burkholderia cepacia complex isolates. (A) Heat map of ANI values among Bcc clinical isolates with type strain of Burkholderia genus. (B) Maximum-likelihood phylogeny of catenated core gene sequences from type strains of Burkholderia genus. [file Image_1.tif]

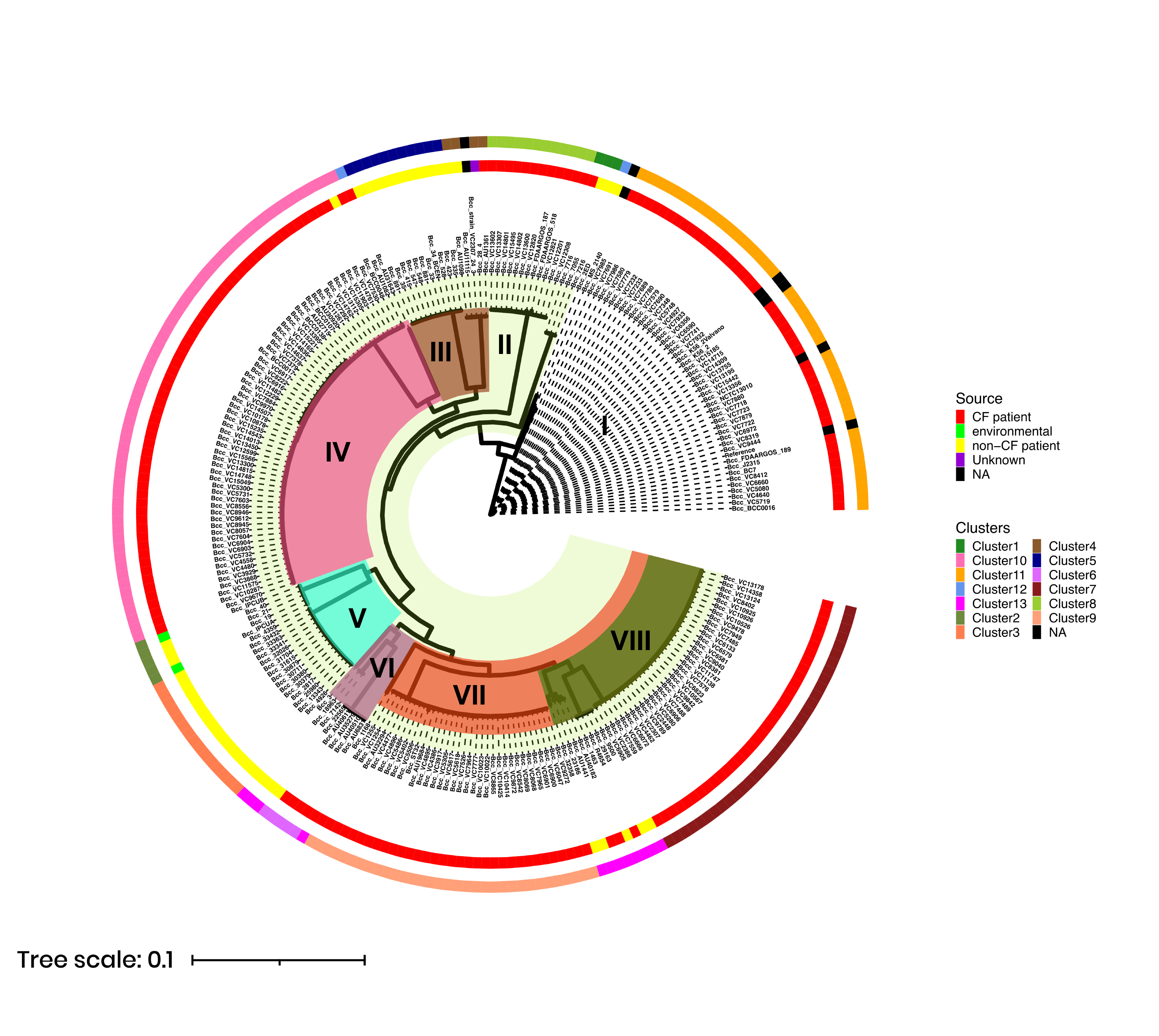

Supplement: Supplementary Figure 2 — Phylogenetic structure of 245 CC31 isolates. Clades I-VIII are marked by background color fill. Data on the source of isolation and fastbaps clusters are depicted on the tree (from inner to outer circle). [file Image_2.tif]

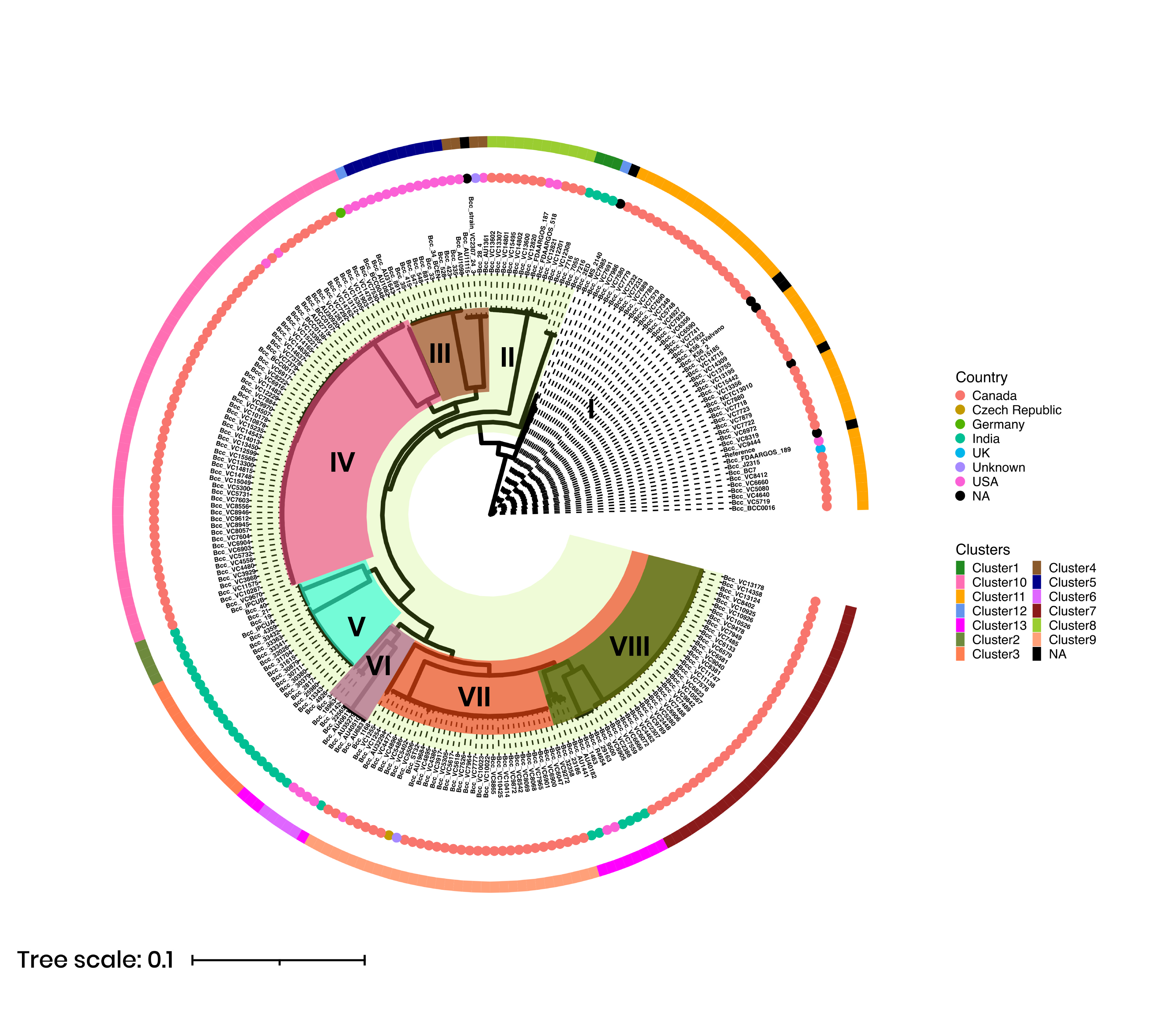

Supplement: Supplementary Figure 3 — Phylogenetic structure of 245 CC31 isolates. Clades I-VIII are marked by background color fill. Data on the country of isolation and fastbaps clusters are depicted on the tree (from inner to outer circle). [file Image_3.tif]

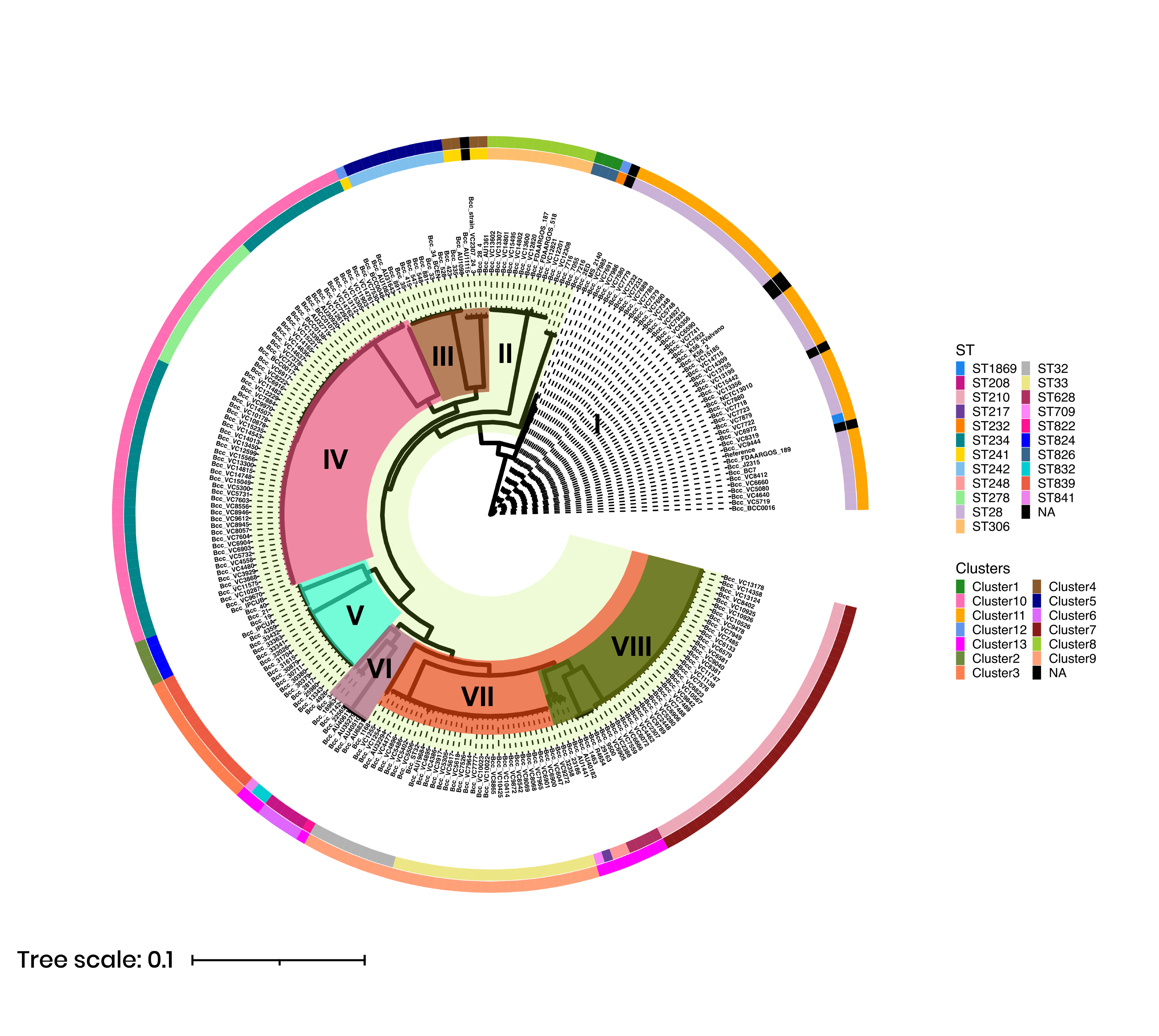

Supplement: Supplementary Figure 4 — Phylogenetic structure of 245 CC31 isolates. Clades I-VIII are marked by background color fill. Data on the MLST classification and fastbaps clusters are depicted on the tree (from inner to outer circle). [file Image_4.tif]

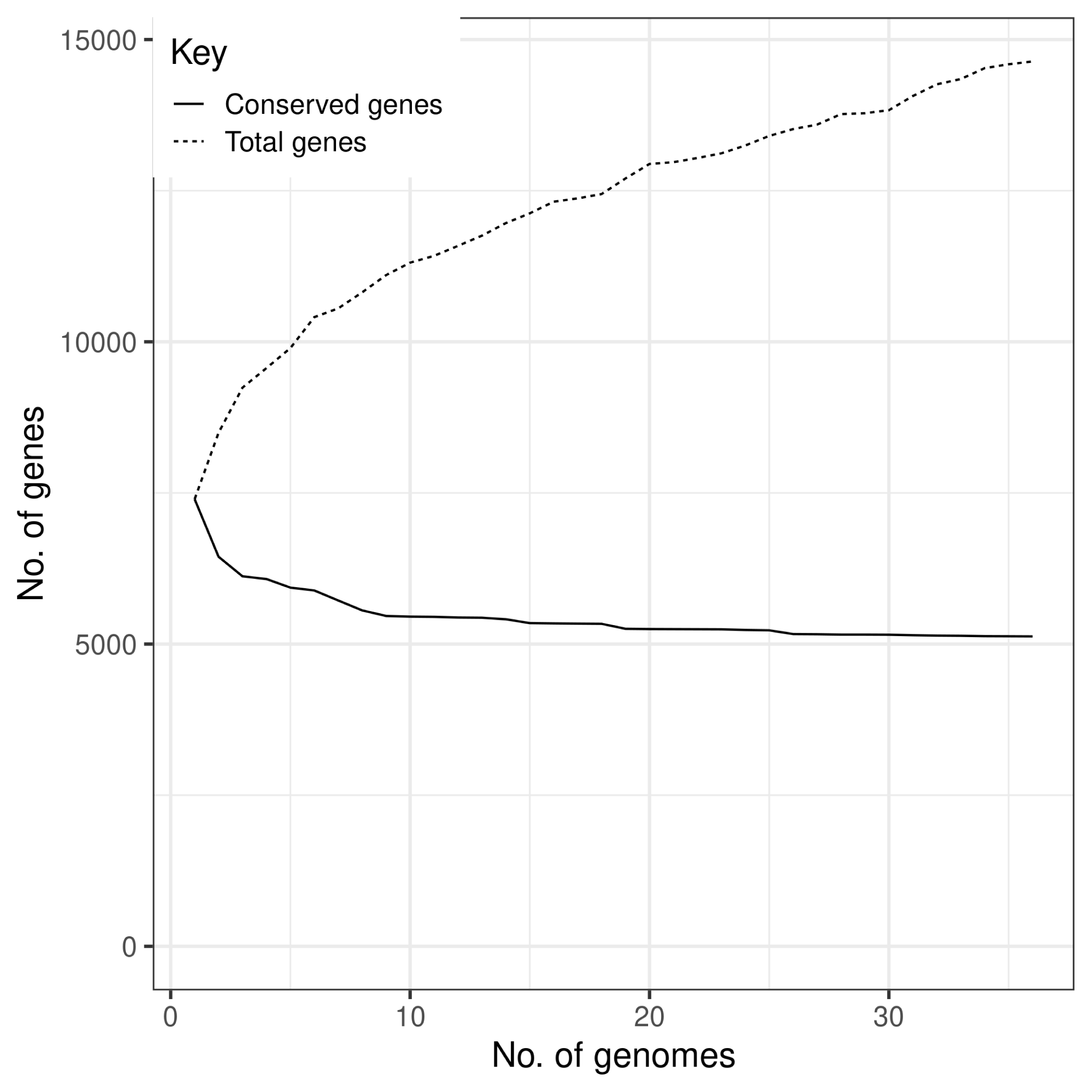

Supplement: Supplementary Figure 5 — Pan-genome curve of B. cenocepacia CC31 isolates. [file Image_5.tif]

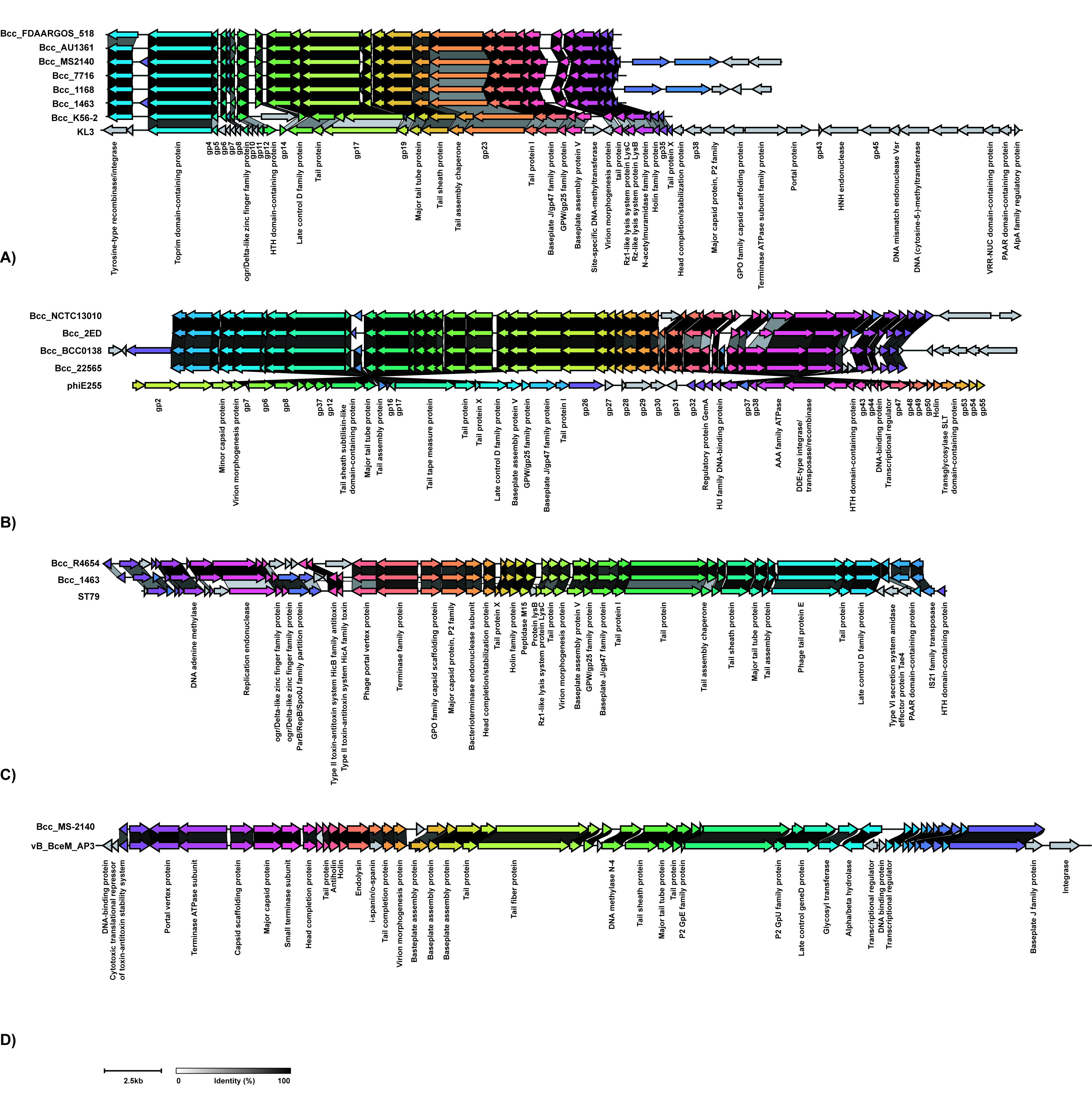

Supplement: Supplementary Figure 6 — Comparison of minor phages KL3 (A), phiE255 (B), ST79 (C), and vB_BceM_AP3 (D) between CC31 isolates across clades. The colored arrowed boxes and labels below boxes represent genes and gene product respectively. Connecting links between genes represent percent identity. The unlabeled arrows represent hypothetical genes. [file Image_6.tif]
